# First nationwide web-based surveillance system for influenza-like illness in pregnant women: participation and representativeness of the French G-GrippeNet cohort

**DOI:** 10.1186/s12889-016-2899-y

**Published:** 2016-03-11

**Authors:** Paul Loubet, Caroline Guerrisi, Clément Turbelin, Béatrice Blondel, Odile Launay, Marc Bardou, Thierry Blanchon, Isabelle Bonmarin, François Goffinet, Pierre-Yves Ancel, Vittoria Colizza, Thomas Hanslik, Solen Kernéis

**Affiliations:** Sorbonne Universités, UPMC Univ Paris 06, INSERM, Institut Pierre Louis d’épidémiologie et de Santé Publique (IPLESP UMRS 1136), F75012 Paris, France; Department of Infectious diseases, CIC Cochin-Pasteur, Cochin Broca Hôtel-Dieu hospital, Assistance Publique-Hôpitaux de Paris (AP-HP), Paris, France; INSERM U 1153, Obstetrical, Perinatal and Pediatric Epidemiology Research Team (EPOPé), Center for Epidemiology and Statistics Sorbonne Paris Cité, DHU Risks in pregnancy, Paris Descartes University, Paris, France; INSERM CIC 1417, Paris, France; Inserm, F-CRIN, Innovative clinical research network in vaccinology (I-REIVAC), Paris, France; Clinical Investigation Center (CIC) INSERM 1432, Gynecology and Obstetrics CIC Network (GO-CIC) & Hepato-gastroenterology department, Dijon Hospital, Dijon, France; Department of Infectious Diseases, Institut de Veille Sanitaire (InVS), St Maurice, France; Department of Obstetrics and Gynecology, Cochin Broca Hôtel-Dieu hospital, Assistance Publique-Hôpitaux de Paris (AP-HP), Paris, France

**Keywords:** Pregnancy, Influenza, Incidence, Surveillance, Web-based study

## Abstract

**Background:**

Pregnancy is a risk factor for severe influenza resulting in increased risks of hospitalisation and death in mothers and their new-borns. Our objective was to assess the representativeness and participation of French women to a new web-based collaborative tool for data collection and monitoring of Influenza Like Illness (ILI) during pregnancy.

**Methods:**

During the 2014/2015 influenza season, pregnant women living in metropolitan France were enrolled through a web platform (https://www.grippenet.fr/). Then throughout the season, participants were asked to report, on a weekly basis, if they had experienced symptoms of ILI. Representativeness was assessed by comparing the characteristics of participants to those of the French National Perinatal Survey. For each participant, the participation rate was the number of weekly questionnaires completed, divided by the length of follow-up (in weeks). Predictors of active participation (participation rate >15 %) were assessed by multivariate logistic regression.

**Results:**

A total of 153 women were enrolled. Participants were older (mean age 34 years vs. 29 years) and more highly educated (high school level 89 % versus 52 %) than the general population of pregnant women in France, but the sample did not differ on pregnancy-related characteristics (parity, history of hospitalisation during a previous pregnancy). The median rate of participation was high (78 %, interquartile range: 34–96). Higher educational level and participation to a previous GrippeNet.fr season were associated with active participation.

**Conclusion:**

Despite small sample size and lack of representativeness, the retention rate was high, suggesting that pregnant women are prone to adhere to a longitudinal follow-up of their health status via the Internet.

## Background

Some infections are more severe in pregnant women and most may also harm the fetus or the new-born. This is the case for influenza that results in higher risks for hospitalisation and death in pregnant women even in the absence of underlying comorbidities [1–3]. Influenza also increases the risks of miscarriage, low birth weight, and prematurity [4–6].

Seasonal influenza surveillance systems provide essential information to describe the diffusion of the epidemics and monitor the incidence through the season [7]. In France, the surveillance is traditionally based on health care systems, excluding people who do not consult for influenza-like illness (ILI) [8, 9]. New technologies and widening of the Internet coverage have opened the way to real-time collaborative data collection and led to the development of innovative approaches for disease surveillance [10, 11]. In 2011, a nationwide surveillance system for ILI, GrippeNet.fr was introduced in France. It is based on the online participation of volunteers from the general population and allows us to gather data on incidence of ILI in the community, and on vaccination coverage, vaccine effectiveness and health-care usage [12, 13]. The participation steadily increased from the first season to reach 6632 participants in 2014/2015.

In pregnant women and women who plan a pregnancy, several web-based studies have already been undertaken, mainly focusing on pre-conception risk factors for adverse pregnancy outcomes [14–18]. Regarding influenza, only one annual transversal web-based survey is conducted each year since 2010/2011 in the USA to assess vaccination coverage among pregnant women [19, 20]. No specific real-time surveillance of ILI in pregnant women is conducted, to our knowledge, and the incidence of the disease is not well documented in this population.

The G-GrippeNet (Grossesse-GrippeNet in French, Pregnancy-GrippeNet) project aimed to describe the epidemiology of seasonal influenza during the influenza season among pregnant women in France. Objectives of the present study were to report data on representativeness of the cohort and factors associated with participation for the opening season 2014/2015.

## Methods

### Study design

The G-GrippeNet project, dedicated to pregnant women, hereafter referred to as GGNET, was integrated into the national GrippeNet.fr web-based annual campaign conducted between November 19th 2014 and April 14th 2015. As described previously [12], GrippeNet.fr (https://www.grippenet.fr/) is an online surveillance project integrated to the European project Influenzanet [21, 22]. Participants are volunteers passively recruited (after communication actions on television, radio, newspapers and others (Influenzanet), poster, and leaflets) in the general population. They register, through their e-mail address, directly on the GrippeNet.fr website. After inclusion, participants are invited to fill in a first questionnaire dealing with general background information. Then, they are invited to self-report on a weekly basis on their health status, reporting easily identifiable symptoms of influenza or stating the absence of symptoms.

### Inclusion criteria

Participants to GGNET had to: 1) be pregnant (regardless of the trimester) 2) be resident in mainland France; 3) read French language; 4) have active email address and Internet access.

### Data collection

Women enrolled were followed throughout the influenza season and asked weekly whether or not they experienced any influenza-like illness. ILI was defined as the combination of the three following criteria: (i) the sudden onset of symptoms, (ii) at least one of the following signs: fever or chills or headache or myalgia or asthenia and (iii) at least one of the following respiratory symptoms: cough, sore throat, shortness of breath (dyspnea) [9].

The intake questionnaire covered age, household size and composition, location of home and workplace, education level, occupation, vaccination status for the previous and present influenza season, the presence of a chronic disease that may lead to more severe influenza case (i.e. chronic respiratory or cardiac diseases, diabetes, and immunosuppression) [12]. Specific questions designed for pregnant women, added to the questionnaire, included pregnancy related factors such as gravidity, parity, estimated date of delivery, history of hospitalisation during a previous pregnancy. Participants were reminded weekly, via an email newsletter, to report through a brief weekly questionnaire (WQ) whether or not they experienced any ILI symptoms since the last seven days. All questionnaires were self-administered. In addition, every month, participants received newsletters on prevention and treatment of infectious diseases during pregnancy, which were used to maintain a communication channel with each participant as a motivation purpose during the season.

### Recruitment of participants

We promoted the project through articles published on web sites dedicated to pregnant women and young mothers’ health. Two national clinical research networks (iREIVAC and GO-CIC), including 30 maternity centres, were also solicited by the investigators. Among them, 10 agreed to participate and promoted the study by displaying advertising posters in their waiting rooms. These 10 maternity centres accounted for 4 % of the total number of delivery per year in the country. No incentives were offered for participation in the study. Consent to participation to the study was obtained through an online form.

### Statistical analysis

Coverage was calculated by dividing the number of participants to GGNET by the average number of pregnant women in France over the study period. The latter was based on national statistics [23], and calculated by summing the number of ingoing pregnancies at the beginning of the study to the number of new pregnancies occurring during the study period. Variables were analysed using Fisher’s exact test or Wilcoxon rank-sum test as appropriate.

To assess the representativeness of our cohort we compared the socio-demographical characteristics of participants of our cohort to those retrieved from the 2010 French National Perinatal Survey [24]. The National Perinatal Surveys are conducted routinely in France and all births occurring during a given week in all maternity units in France are included. Women are interviewed between delivery and discharge about their socio-demographic characteristics, prenatal care and health behaviour. Data on health and obstetric care are collected from medical records. The sample is representative of all births at a national level [24].

To evaluate the participation to the study, two indicators: participation rate and percentage of “active participants” were calculated and compared to those of other (non-pregnant) participants to the general GrippeNet.fr cohort. For each participant, the participation rate was the number of WQ completed, divided by the length of follow-up (in weeks). The follow-up started at inclusion (registration on the platform) and ended at delivery. Participants were considered as “active participants” if their participation rate was above 15 % (definition used in previous GrippeNet.fr and Influenzanet studies [12, 22, 25]). Indicators of participation were estimated in our sample and compared to those calculated among non-pregnant participants to the general cohort of GrippeNet.fr. Factors associated with active participation within the GGNET cohort were assessed using a multivariate logistic regression model. We first tested all sociodemographics and pregnancy-related variables in univariate analyses. Second, we entered explicative variables with a p-value <0.20 in a multivariate model. We then used a backward stepwise selection procedure (removal criteria: *p* > 0.05) to built the final model. Finally, the variable « participation to a previous season of GrippeNet.fr”, which was a known factor of higher active participation in literature [12, 26], was added to the model as an adjustment variable. The results of both analyses are presented.

Statistical analysis was performed using R software (version 3.2) [27].

### Privacy and ethical approval

Data were anonymous and participants were free to withdraw from the study at any time. This study was conducted in agreement with French regulations on privacy, data collection and treatment and was approved by the Comité consultatif sur le traitement de l’information en matière de recherche (CCTIRS, Advisory committee on information processing for research, authorisation 11.565) and by the Commission Nationale de l’Informatique et des Libertés (CNIL, French Data Protection Authority, authorisation DR-2012–024).

## Results

### Participants

Over the study period - November 19th 2014 and April 14th 2015–153 women were enrolled among 780 000 eligible (coverage: 20 individuals per 100,000 pregnant women). Participation was widespread in the country, with participants coming from 53 out of 96 mainland French departments. Overall, mean age was 34 years (standard deviation, SD 4.3). One hundred thirty five (88 %) had a university degree and 111 (74 %) were employed at the time of the study. Forty (27 %), 48 (32 %) and 61 (41 %) women were respectively in their first, second and third trimester of pregnancy. Nulliparity concerned 73 participants (48 %). Thirty-eight participants (25 %) gave birth during the influenza period. (Table 1)

**Table 1:** Comparison of sample’s characteristics of G-GrippeNet 2014/2015 with national statistics from the 2010 National Perinatal Survey

	GGNET *N* (%)	National perinatal survey 2010 *N* (%)	*p*-value
Total	153	15 187	
Age, years			*<10* ^*−3*^
< 25	1 (1 %)	2444 (17 %)	
25–29	29 (19 %)	4777 (33 %)	
30–34	59 (39 %)	4419 (31 %)	
35–39	51 (34 %)	2255 (16 %)	
> 39	12 (8 %)	506 (3 %)	
Educational Level			*<10* ^*−3*^
Middle School or less	3 (2 %)	3974 (28 %)	
High School	13 (9 %)	2796 (20 %)	
College or more	135 (89 %)	7290 (52 %)	
Occupational status			*<10* ^*−3*^
Employed	111 (74 %)	9507 (67 %)	
Housewife	5 (2 %)	1869 (13 %)	
Student	1 (1 %)	344 (2 %)	
Unemployed	14 (9 %)	1711 (12 %)	
Other	21 (14 %)	718 (5 %)	
Occupation (if employed)			*<10* ^*−3*^
Professional, manager, intermediate	36 (31 %)	4175 (43 %)	
Office work	64 (55 %)	2714 (29 %)	
Services	5 (4 %)	1713 (18 %)	
Skilled manual work	6 (5 %)	453 (5 %)	
Other manual work	0	339 (4 %)	
Other	6 (5 %)	88 (1 %)	
Pregnancy trimester			*-*
1st	40 (27 %)	-	
2nd	47 (32 %)	-	
3rd	61 (41 %)	-	
Parity			*0.45*
0	73 (48 %)	6396 (43 %)	
1	53 (35 %)	5004 (35 %)	
2	20 (13 %)	2069 (14 %)	
> 2	7 (4 %)	1130 (8 %)	
History of hospitalisation during previous pregnancy			*-*
No	82 (78 %)	-	
Yes	23 (22 %)	-	
Chronic disease			
No	142 (93 %)	-	-
Yes	11 (7 %)	-	
Asthma	7	-	
Chronic respiratory disease	2	-	
Chronic cardiac disease	1	-	
Chronic renal disease	1	-	
Diabetes	0	-	
Immunosuppression	1	-	
Body Mass Index before pregnancy			*0.25*
< 18.5	11 (7 %)	1126 (8 %)	
18.5–25	106 (70 %)	8811 (65 %)	
25–30	26 (17 %)	2360 (17 %)	
> 30	8 (6 %)	1347 (10 %)	
Current smoker			*0.002*
No	142 (93 %)	11,679 (83 %)	
Yes (1)	11 (7 %)	2403 (17 %)	

(1) at enrollment in GGNET; during the 3rd trimester in the National Perinatal Survey

### Representativeness

Compared to the general population of French pregnant women, participants of the GGNET cohort were older (mean age 34.0 years (SD 4.3) vs. 29.7 years (SD 5.3)), more highly educated, more frequently employed and less frequently smokers (Table 1). On the other hand, no difference was found on Body Mass Index (BMI), parity or history of hospitalisation during a previous pregnancy.

### Participation

During the study period, 152 pregnant women filled in at least one WQ, and a total of 1363 WQ were completed. The mean time of follow-up was 9.0 weeks (SD 6.6). The median rate of participation was 78 % (Inter Quartile Range IQR, 34–96) and 128 (84 %) participants were considered as ‘active participants’.

Compared to the other participants of GrippeNet.fr, the median rate of participation in pregnant participants to GGNET did not differ (78 % (IQR, 34–96) vs. 87 % (IQR, 45–96) respectively, p-value 0.37). Likewise, the percentage of active participants did not differ between the two cohorts (84 % vs. 84 %, GGNET vs. GrippeNet.fr, respectively, p-value 0.92). (Table 2)

**Table 2:** Comparison of participation between pregnant participants of G-GrippeNet and general participants of GrippeNet.fr 2014/2015

	GGNET	General Participants	*p-value*
Number of participants	153	6 574	
Number of weekly questionnaire	1 363	89 172	
Mean number of weekly questionnaires	9	13	
Median rate of participation (IQR)	78 % (34 – 96 %)	87 % (45 – 96 %)	0.37
Active participants (%)	128 (84 %)	5504 (84 %)	0.92

Educational level and occupation were selected for the first step of the multivariate analysis of predictors of active participation. Participation to a previous GrippeNet.fr season was then included in the second step of the procedure. At the end of the model selection procedure, although not significant in the multivariate model, higher educational level and previous participation tended to be associated with an active participation (Odds Ratio, OR 2.52, IC95% 0.70–8.11 and OR 2.93, IC95% 0.90–13.27, respectively). (Table 3)

**Table 3:** Rate of active participant in the 2014/2015 G-GrippeNet cohort depending on characteristics, and factors associated with active participation (= participation rate > 15 %) in univariate and multivariate logistic regression

	*N* (%)	OR [95 % CI]^a^	*p*-value	ORa [95 % CI]**	*p*-value	ORa [95 % CI]***	*p*-value
Total of active participants	128/153 (84 %)	-	-	-	-	-	-
Age, years							
< 35	60/74 (82 %)	1	*0.32*	*-*	*-*	*-*	*-*
35 +	67/77 (87 %)	1.56[0.65–3.88]		*-*		*-*	
Educational Level							
Less than High school	11/16 (69 %)	1	*0.09*	1	*0.15*	1	*0.13*
High school or more	116/135 (86 %)	2.77[0.80–8.59]		2.41[0.68–7.68]		2.52[0.70–8.11]	
Occupation							
Professional	28/32 (88 %)	1	*0.09*	1	*0.13*	1	*0.19*
Office work	56/62 (90 %)	1.33[0.32–5.05]		1.41[0.34–1.61]		1.21 [0.28–4.75]	
Other	44/58 (76 %)	0.45[0.12–1.40]		0.51[0.13–1.61]		0.53 [0.14–1.69]	
Pregnancy trimester							
1st & 2nd	72/87 (83 %)	1	*0.68*	*-*	*-*	*-*	*-*
3rd	52/61 (85 %)	1.20[0.50–3.06]		*-*		*-*	
Parity							
0	63/73 (86 %)	1	*0.49*	*-*	*-*	*-*	*-*
≥ 1	65/79 (82 %)	0.74[0.30–1.77]		*-*		*-*	
Chronic disease							
No	120/142 (85 %)	1	*0.71*	*-*	*-*	*-*	*-*
Yes	8/10 (80 %)	0.73[0.17–5.10]		*-*		*-*	
Previous participation to GrippeNet.fr							
No	86/107 (80 %)	1	*0.03*	*-*	*-*	*1*	*0.10*
Yes	42/45 (93 %)	3.41[1.12–15.05]		-		2.93 [0.90–13.27]	

^a^Univariate analysis

**Multivariate analysis including all factors associated with active participation in univariate analysis (*p* < 0.2). (Model 1)

***Multivariate analysis including all factors associated with active participation in model 1 (*p* < 0.05) + previous participation to GrippeNet.fr

## Discussion

GGNET is the first nationwide web-based influenza surveillance system specifically targeting pregnant women. For the first season in 2014/2015, 153 pregnant women were enrolled. Participants were distributed in more than half of French departments, differently from previous birth-cohorts approaches that were mainly local [18]. However, the sample was small (reaching a coverage of 20/100 000) and non-representative. Pregnant women of our cohort were older, with a higher degree of education and more frequently employed than the general population of pregnant women in France. Active participation was high and similar to that observed in participants of the general GrippeNet.fr cohort.

Only a few web-based studies have been conducted so far in the pregnant population. Most of them were large transversal studies assessing women behaviour or effects of interventions [16, 18, 28–30]. To our knowledge, no web-based survey has offered a longitudinal follow-up on a weekly basis. The strengths of our study are: a wide geographical distribution of participants at a national scale, and a high active participation rate. The latter being probably related to the simplicity of follow-up (taking only few minutes per week with weekly automatic email reminders).

Main limitations are the small sample size and the non-representativeness of the sample. Despite our efforts, we calculated that our sample represented only 0.02 % of French women who were pregnant during the study period. Our communication campaign was based on both online (web sites dedicated to pregnant women and young mothers’ health) and offline methods (advertising posters displayed in waiting rooms of large maternity centres). Among women who agreed to participate, half reported to have heard about the study via the Internet and only 15 % through health care workers. This suggests that the Web and associated online media (e.g. mobile phone app) are of crucial importance to assemble a large cohort and ensure high participation for the next influenza seasons. Moreover, previous work has shown that in France, contrary to what observed in other Influenzanet countries, participants recruited through online communication have a higher follow-up participation [31]. The lack of representativeness of our sample, likely induced by the non-representative nature of the Internet population, thus translating into coverage biases, and the self-selection of participants, also called the ‘volunteer effect’ (i.e. those who choose to volunteer for studies may differ in lifestyle and health from those who decline) [11] was similarly observed for the entire Grippenet.fr cohort in comparison to the general population. In previous GrippeNet.fr seasons, other authors reported a large underrepresentation of the youngest and oldest age classes in the participants compared to the French population [12, 25]. These findings were also reported in web-based birth cohorts [18]. This underlines that the next communication campaigns should preferentially target younger pregnant women. Again, the use of new, Internet-based communication channels is likely to have the largest impact in this population.

In this study, the active participation rate was high and factors that we found associated with active participation in multivariate analysis are in line with previous research that has shown that different factors can influence participation rate. Socio-demographic factors, such as low educational level or unemployment, as well as unhealthy life style factors (smoking, alcohol consumption) are typically related to increased risk of non-response and attrition in epidemiological studies [32, 33]. This was also observed in a large study on the European Influenzanet cohort [26]. Longitudinal studies are important in public health research for identifying risk factors related to negative health outcomes. However, a major concern in such studies is that the longer the follow-up period, the higher are the chances for dropout. In our study, active participation of pregnant women was similar to that observed in participants of the general GrippeNet.fr cohort.

Social medias and widening of the Internet coverage have opened the way to real-time collaborative data collection and led to the development of innovative approaches for disease surveillance. This longitudinal web-based surveillance study proved feasible in pregnant women and will bring original data on the epidemiology of influenza in this population. It will be helpful to promote vaccination in this population.

## Conclusions

We report the results of the first prospective cohort study aiming to describe influenza epidemic in pregnant women. Despite the small sample size and lack of representativeness on certain demographic variables, the active participation rate was high, suggesting that pregnant women are prone to adhere to a longitudinal follow-up of their health status via the Internet. In multivariate analysis, higher educational level and previous participation tended to be associated with an active participation. To reach younger participants with mixed educational level, it also seems crucial, besides traditional channels, to encourage enrolment via online social media sites and apps.
